# Integrated Profiles Analysis Identified a Coding-Non-Coding Signature for Predicting Lymph Node Metastasis and Prognosis in Cervical Cancer

**DOI:** 10.3389/fcell.2020.631491

**Published:** 2021-01-21

**Authors:** Yu Zhang, Di Sun, Jiayu Song, Nan Yang, Yunyan Zhang

**Affiliations:** ^1^Department of Radiotherapy, Harbin Medical University Cancer Hospital, Harbin, China; ^2^Department of Radiation Therapy Technology Center, Harbin Medical University Cancer Hospital, Harbin, China; ^3^Department of Gynecological Radiotherapy, Harbin Medical University Cancer Hospital, Harbin, China

**Keywords:** cervical cancer, lymph node metastasis, machine learning, signature, biomarker

## Abstract

Accumulating evidence has shown that lymph node metastasis (LNM) is not only an important prognostic factor but also an indicator of the need for postoperative chemoradiotherapy. Therefore, identifying risk factors or molecular markers related to LNM is critical for predicting the prognosis and guiding individualized treatment of patients with cervical cancer. In this study, we used the machine learning-based feature selection approach to identify eight optimal biomarkers from the list of 250 differentially expressed protein-coding genes and long non-coding RNAs (lncRNAs) in the TCGA cohort. Then a coding-non-coding signature (named CNC8SIG) was developed using the elastic-net logistic regression approach based on the expression levels of eight optimal biomarkers, which is useful in discriminating patients with LNM from those without LNM in the discovery cohort. The predictive performance of the CNC8SIG was further validated in two independent patient cohorts. Moreover, the CNC8SIG was significantly associated with patient’s survival in different patient cohorts. *In silico* functional analysis suggested that the CNC8SIG-associated mRNAs are enriched in known cancer-related biological pathways such as the Wnt signaling pathway, the Ras signaling pathway, Rap1 signaling pathway, and PI3K-Akt signaling pathway.

## Introduction

Cervical cancer is the second leading cause of cancer death in women aged 20 to 39 years. There was an estimated new cervical cancer case of 13800 and an estimated death of 4290 in the United States in 2020 (Siegel et al., 2020). Lymph node metastasis (LNM) status has been reported to be one of the most important prognostic factors and is significantly related to the clinic-pathologic characters (Du et al., 2018). Although radical hysterectomy followed by pelvic lymphadenectomy is the standard surgical management for patients with early-stage cervical cancer, pelvic lymphadenectomy may be unnecessary for most patients with early-stage cervical cancer with low risk of LNM. Furthermore, LNM status also is an indicator of the need for postoperative radiotherapy. Therefore, identifying risk factors or molecular markers related to LNM is critical for predicting the prognosis and guiding individualized radiotherapy of patients with cervical cancer.

With the development and advances in high throughput sequencing such as microarray and RNA sequencing (RNA-Seq) technologies, gene expression-based markers have been widely identified and applied in a variety of cancers. In cervical cancer, gene expression profiles have been analyzed to identify critical genes and pathways involved in cancer development and progression in many previous studies (Wong et al., 2006; Wu et al., 2018; Yang et al., 2020). Some gene signatures were also developed to predict prognosis and recurrence for aiding clinical decisions. For example, Xie et al. (2019) identified an 8-gene signature to predict the prognosis of patients with cervical cancer following radiotherapy by analyzing matched gene expression profiles and DNA methylation profiles. Recently, Nguyen identified a 70-gene signature for predicting the therapy outcome and choosing patients who benefit from molecular-targeted therapy in advanced-stage cervical cancer (Nguyen et al., 2020). Zhao et al. (2020) developed a 5-gene prognostic model to predict a patient’s overall survival. Although several gene signatures have been developed to predict LNM status, these existing predictive signatures mainly focused on protein-coding genes. Increasing evidence has suggested that long non-coding RNAs (lncRNAs) play crucial roles in the progression, invasion, and metastasis of cervical cancer (He et al., 2020; Zhong et al., 2020), therefore implying the potential of dysregulated lncRNAs as novel biomarkers in predicting LNM status and prognosis.

In this study, we performed integrative analysis for mRNA expression profiles and lncRNA expression profiles in a large cohort of patients with cervical cancer and used a machine learning approach to identify novel coding-non-coding RNA signature for predicting LNM status and prognosis.

## Materials and Methods

### Cervical Cancer Patient Datasets

Level-3 RNA-sequencing data (HTSeq-Counts and HTSeq-FPKM) and clinical information of 193 cervical cancer patients with lymph node metastasis information were obtained from UCSC Xena^1^. The microarray data (Affymetrix Human Genome U133 Plus 2.0 Array) and clinical information of 39 cervical cancer patients with lymph node metastasis information were downloaded from the Gene Expression Omnibus (GEO) database^2^. Another independent cohort of 300 cervical cancer patients with survival information was obtained from the GEO database^3^.

### Acquisition and Analysis of lncRNA and mRNA Expression Profiles

GTF files (GRCH38) were downloaded from The Encyclopedia of DNA Elements (GENCODE)^4^. For the TCGA cohort, according to the GTF files and previously described, we obtained 14212 lncRNAs with biotype of 3prime_overlapping_ ncRNA, antisense, bidirectional_promoter_ lncRNA, lincRNA, macro_ lncRNA, non_coding, processed_transcript, sense_ intronic, sense_ overlapping, and 19645 mRNAs with the “protein_ coding” biotype. For the GEO cohort, raw microarray data (CEL files) profiled from Affymetrix Human Genome U133 Plus 2.0 Array were obtained from the GSE26511 and were processed and normalized using the Robust Multichip Average (RMA) algorithm for background subtraction, quantile normalization and summarization. By repurposing array probes into the human genome (GRCh 38) and GENCODE database, a total of 6254 lncRNAs and 18652 mRNAs were obtained. To perform cross-validation analysis among different cohorts based on different platforms, 6254 overlapped lncRNA and 18652 overlapped mRNAs among different platforms were kept for further analysis.

Differential expression analyses of lncRNAs and mRNAs between cervical cancer patients with and without lymph node metastasis were performed using the R package “DESeq2” (version 1.24.0) (Ritchie et al., 2015). Those lncRNAs and mRNAs with |log2(fold change)| > 1 and false discovery rate (FDR) *p*-value < 0.05 were identified as differentially expressed lncRNAs and mRNA.

### Construction of the Predictive Model

Because of the imbalance of the samples with and without lymph node metastasis, we first used Synthetic Minority Over-sampling TEchnique (SMOTE) method with R package “DMwR” to overcome imbalances by generating artificial data based on feature space similarities from minority samples in the original training cohort (Alghamdi et al., 2017). Then we used random forest-recursive feature elimination (RF-RFE) approach with 10-fold cross-validation and five re-sampling as the feature selection method for differentially expressed mRNAs and lncRNAs to identify significant features associated with lymph node metastasis and for further use in the predictive model. Finally, an elastic-net logistic regression approach was used to generate a classifier for predicting lymph node metastasis.

### Functional Enrichment Analysis

Functional enrichment analysis was performed on Gene Ontology (GO) and Kyoto encyclopedia of genes and genomes (KEGG) pathway to infer possible biological function in the Database for Annotation, Visualization, and Integrated Discovery (DAVID)^5^ are limited in the GO biological biology (BP) terms and KEGG pathways (Huang da et al., 2009a, b). Those GO terms and KEGG pathways with *p* < 0.05 significantly enriched GO terms and KEGG pathways using functional annotation chart options with the whole human genome as background as described in previous studies (Zhou et al., 2018; Bao et al., 2020).

### Statistical Analysis

Kaplan-Meier survival curves and log-rank tests were used to assess the differences in survival time between different patient groups with the R packages “survival” (v2.44-1.1) and “survminer” (v0.4.6). Hierarchical clustering was performed with Euclidean distance and complete linkage (Sun et al., 2020; Zhou et al., 2020). The predictive performance of the signature was evaluated using the receiver operating characteristic (ROC) analysis and the area under the curve (AUC) value was calculated with the R package “pROC.” All statistical analyses were performed using R software and Bio-conductor.

## Results

### Identification of Key mRNAs and lncRNAs Associated With Lymph Node Metastasis

**FIGURE 1:**
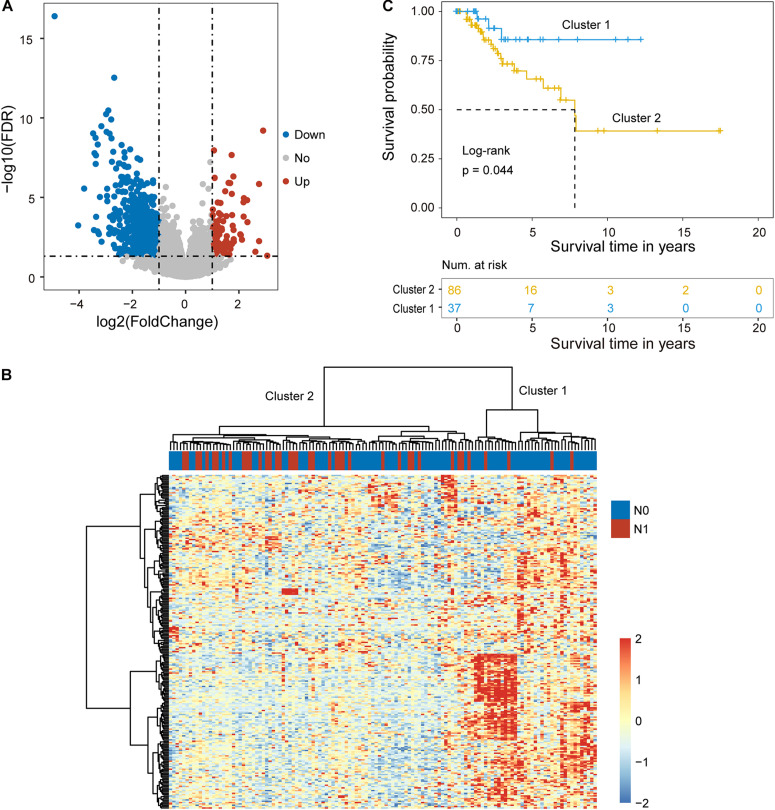
Identification of candidate biomarkers related to lymph node metastasis. **(A)** Volcano plot displaying differentially expressed mRNAs and lncRNAs between cervical cancer patients with and without lymph node metastasis. **(B)** Unsupervised clustering of patients based on the expression levels of differentially expressed mRNAs and lncRNAs. **(C)** Kaplan-Meier survival curves of patients in cluster 1 and cluster 2.

One hundred ninety-three cervical cancer patients with lymph node metastasis information were divided into the training cohort (*n* = 129; 89 patients without LNM and 40 patients with LNM) and testing cohort (*n* = 64; 44 patients with LNM and 20 patients without LNM) according to a 2:1 ratio. To identify key mRNAs and lncRNAs associated with lymph node metastasis, we first compared the mRNA and lncRNA expression profiles obtained from 40 patients that were metastatic to lymph nodes (N+) to 89 patients that were not (N−) in the training cohort. A total of 224 mRNAs and 26 lncRNAs were identified as differently expressed mRNAs and lncRNAs (| log2(fold change)| > 1 and FDR *p*-value < 0.05) (Figure 1A). Among them, 28 mRNAs and two lncRNAs were observed to be upregulated, and 196 mRNAs and 24 lncRNAs were downregulated in cervical cancer patients with lymph node metastasis compared to those that were not (Supplementary Table 1).

Hierarchical clustering of the expression values of differentially expressed mRNAs and lncRNAs for 89 patients in the training cohort produced two distinctive patients’ clusters with significantly different lymph node metastasis status (Figure 1B). Furthermore, there was a significant difference in overall survival time between the LNM-like group and the non-LNM-like group (log-rank *p* = 0.044) (Figure 1C). Patients in the LNM-like group tended to have a significantly poor prognosis compared to those in the non-LNM-like group (median 7.83 years vs. NA) (Figure 1C).

### Construction of an Integrative Coding-Non-Coding Signature for Predicting Lymph Node Metastasis

**FIGURE 2:**
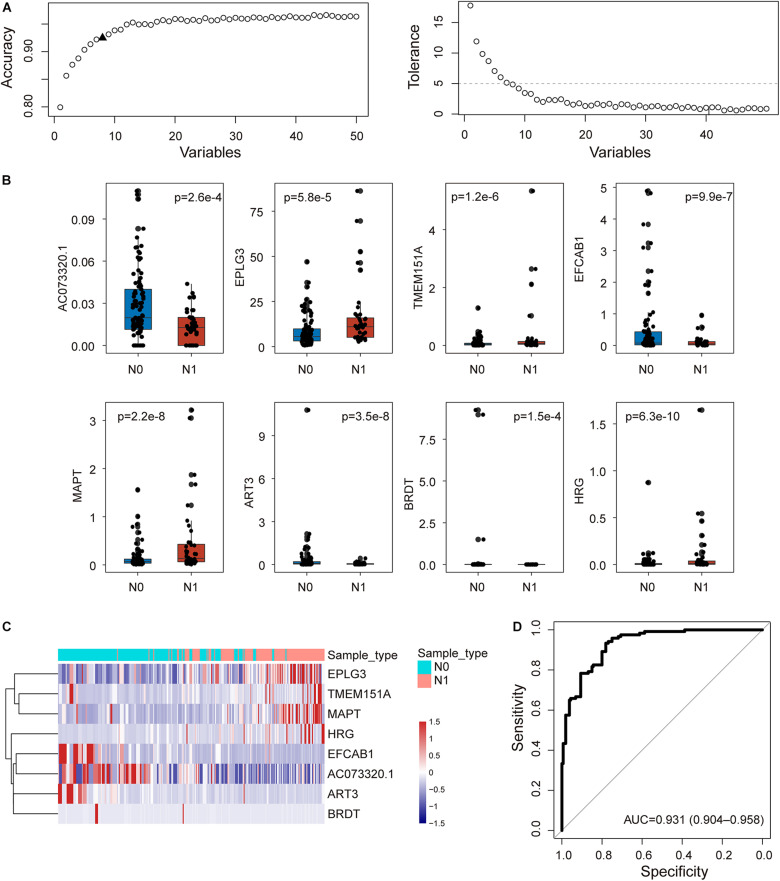
Development of the coding-non-coding RNA signature in the discovery cohort. **(A)** The predicted accuracy of each combination is constructed by a specific number of candidate biomarkers. **(B)** Boxplots displaying expression pattern of eight biomarkers. **(C)** Unsupervised clustering of patients based on the expression levels of eight biomarkers. **(D)** Receiver operating characteristic (ROC) curves for the coding-non-coding RNA signature in predicting lymph node metastasis.

To construct an integrative coding-non-coding signature for predicting lymph node metastasis, we performed machine learning-based feature selection for differently expressed 224 mRNAs and 26 lncRNAs using RF-RFE approach with 10-fold cross-validation and five re-sampling (Figure 2A). Finally, eight optimal features, including seven mRNAs (*EPLG3*, *TMEM151A*, *EFCAB1*, *MAPT*, *ART3*, *BRDT*, and *HRG*) and one lncRNAs (*AC073320.1*), were selected for constructing predictive model when considering the balance between performance and number of the signature (Table 1). Then, a coding-non-coding signature (CNC8SIG) was developed based on the expression levels of seven mRNAs and one lncRNA using the elastic-net logistic regression approach. Among the CNC8SIG, four genes, including *EPLG3*, *TMEM151A*, *MAPT*, and *HRG* were up-regulated in patients with LNM, whereas the other four genes (*AC073320.1*, *EFCAB1*, *ART3*, and *BRDT*) tended to be down-regulated in patients with LNM (Figures 2B,C). When the CNC8SIG was tested in the training cohort, ROC analysis revealed that the CNC8SIG achieved an AUC value of 0.931 (0.904–0.958) with an accuracy of 83.6%, sensitivity of 79.2% and specificity of 86.9%, as showed in Figure 2D.

**TABLE 1:** Detailed information of eight biomarkers in the coding-non-coding RNA signature.

Ensembl id	Gene name	Type	Genomic location
ENSG00000143590	*EPLG3*	mRNA	Chr1: 155,078,837-155,087,538(+)
ENSG00000179292	*TMEM151A*	mRNA	Chr11: 66,291,894-66,296,664(+)
ENSG00000034239	*EFCAB1*	mRNA	Chr8: 48,710,789-48,735,311(−)
ENSG00000186868	*MAPT*	mRNA	Chr17: 45,894,551-46,028,334(+)
ENSG00000244036	*AC073320.1*	lncRNA	Chr7: 129,953,234-130,026,989(+)
ENSG00000156219	*ART3*	mRNA	Chr4: 76,011,184-76,112,802(+)
ENSG00000137948	*BRDT*	mRNA	Chr1: 91,949,371-92,014,426(+)
ENSG00000113905	*HRG*	mRNA	Chr 3: 186,660,216-186,678,234(+)

### Independent Validation of the Predictive Performance of the CNC8SIG in Different Cohorts

**FIGURE 3:**
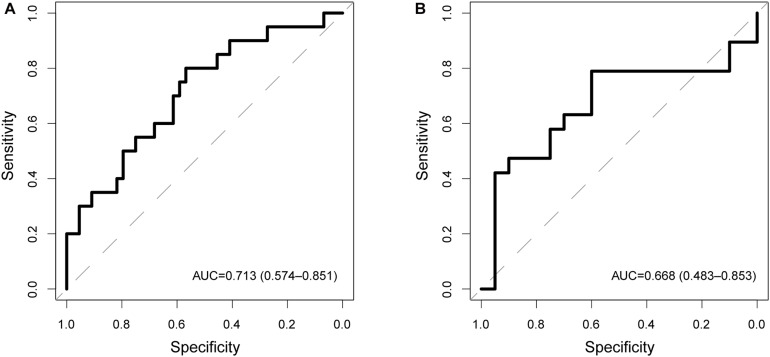
Independent validation in different patient cohorts. Receiver operating characteristic (ROC) curves for the coding-non-coding RNA signature in predicting lymph node metastasis in the testing cohort **(A)** and GSE26511 cohort **(B)**.

To examine the robustness and reproducibility of the CNC8SIG for predicting lymph node metastasis, we further tested the CNC8SIG in the other two patient cohorts. We first applied the CNC8SIG to 64 patients in the testing cohort. As showed in Figure 3A, ROC analysis revealed that the CNC8SIG achieved an AUC value of 0.713 (0.574–0.851) with an accuracy of 68.8%, sensitivity of 45% and specificity of 79.6%. Further validation of the CNC8SIG was performed on the completely independent GEO GSE26511 cohort. As shown in Figure 3B, the CNC8SIG also revealed well-predicted performance for lymph node metastasis with an AUC value of 0.66 (0.483–0.853) (Figure 3B). The above results from different patient cohorts confirmed the robustness and reproducibility of the CNC8SIG for predicting lymph node metastasis.

### Association of the CNC8SIG With Patients’ Prognosis

**FIGURE 4:**
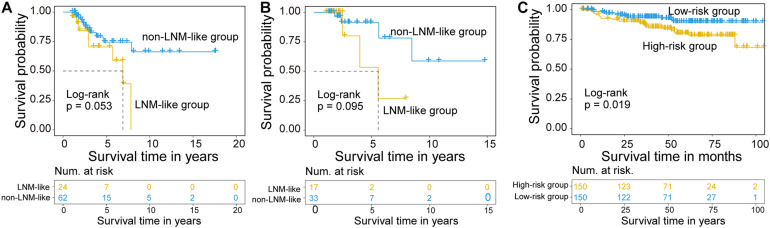
Association of the coding-non-coding RNA signature with prognosis. Kaplan-Meier survival curves of patients between different patient groups in the discovery cohort **(A)**, testing cohort **(B)**, and GSE44001 **(C)**.

We further examine the association between the CNC8SIG and patients’ prognosis by comparing the survival time between the non-LNM-like patient group and LNM-like patient group predicted by the CNC8SIG using the Kaplan-Meier curves and log-rank test. In the training cohort, as shown in Figure 4A, the non-LNM-like patient group predicted by the CNC8SIG had a significantly better prognosis than the predicted LNM-like patient group (median NA vs. 6.9 years; log-rank *p* = 0.053) (Figure 4A). In the testing cohort, survival analysis revealed that the non-LNM-like patient group predicted by the CNC8SIG tended to have a long good prognosis compared to the predicted LNM-like patient group (median NA vs. 5.57 years; log-rank *p* = 0.095) (Figure 4B). When the CNC8SIG was tested in the GSE44001, the CNC8SIG stratified 300 patients into a high-risk group and low-risk group with significantly different survival times. As shown in Figure 4C, patients in the high-risk group have a poor prognosis compared to those in the low-risk group (log-rank *p* = 0.019) (Figure 4C). These results demonstrated the association of the CNC8SIG with a patient’s prognosis.

### *In silico* Functional Analysis for the CNC8SIG

**FIGURE 5:**
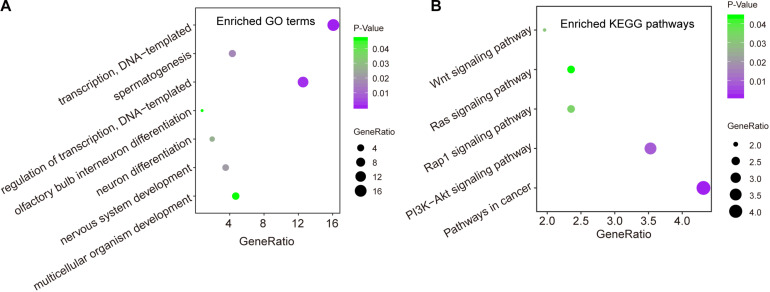
*In silico* functional analysis of the coding-non-coding RNA signature. **(A)** Enriched GO terms. **(B)** Enriched KEGG pathways.

We first measured the expression levels of lncRNA *AC073320.1* with other mRNAs using the Pearson correlation coefficient and identified 253 mRNAs correlated with *AC073320.1* (Pearson correlation coefficient > 0.3 and *p* < 0.05). Then we performed functional enrichment analysis for *AC073320.1*-associated 253 mRNAs and other seven mRNAs in the CNC8SIG. GO analysis identified seven enriched biological processes mainly involved in transcription regulation, organism development and differentiation (Figure 5A). KEGG enrichment analysis revealed that the CNC8SIG-associated mRNAs are enriched in known cancer-related biological pathways such as the Wnt signaling pathway, Ras signaling pathway, Rap1 signaling pathway and PI3K-Akt signaling pathway (Figure 5B).

## Discussion

Cervical cancer is the second leading cause of cancer death in women. LNM is well known as one of the important factors in the International Federation of Gynecology and Obstetrics (FIGO) staging system for guiding precision treatment and management of cervical cancer. Furthermore, it was also reported that LNM was not only closely associated with prognosis but also with treatment planning. Patients with LNM may benefit from chemoradiotherapy rather than surgery as their first choice (Wu et al., 2020). Therefore, the diagnosis of LNM in cervical cancer is critical for guiding individualized treatment and avoiding unnecessary surgical intervention. However, the traditional methods, such as magnetic resonance imaging (MRI), have limited sensitivity in diagnosing LNM in cervical cancer. Therefore, it is urgently needed to identify novel molecular markers related to LNM for predicting the prognosis and guiding individualized treatment of patients with cervical cancer.

In this study, we performed integrative analysis for mRNA expression profiles and lncRNA expression profiles in a large cohort of cervical cancer patients with and without LNM, and identified 224 mRNAs and 26 lncRNAs as candidate biomarkers. Then we applied a machine learning approach for feature selection for these candidate biomarkers and identified eight optimal biomarkers, including seven mRNAs and one lncRNAs. To accelerate clinical application, we used an elastic-net logistic regression approach to develop a predictive signature based on the expression levels of eight optimal biomarkers (named CNC8SIG). The CNC8SIG revealed very well predictive performance in discriminating patients with LNM from those without LNM in the discovery cohort. The CNC8SIG also exhibited differentiated performance in determining the LNM status in other independent patient cohorts. Furthermore, survival analysis revealed that different risk patient groups have significantly different survival outcomes in different patient cohorts. These results suggested that the CNC8SIG not only has very well predictive performance for lymph node metastasis but also is associated with patients ‘prognosis.

In the CNC8SIG, several predictive genes have been reported to be associated with cancer development and progression. Previous studies have reported that overexpression of *ART3* could increase cell proliferation, invasion of triple-negative breast cancer cells via activation of Akt and ERK pathways, and the dysregulation of *ART3* was significantly associated with survival (*ART3* regulates triple-negative breast cancer cell (Tan et al., 2016). *BRDT* is an important member of Bromodomain and extraterminal domain (BET) family (Wan et al., 2020). Histidine-rich glycoprotein (*HRG*) has been reported to have a wide array of functions, such as immunity, cell adhesion, angiogenesis, and thrombosis (Johnson et al., 2014). Aberrant expression of *HRG* has been implicated in several cancers. For example, decreased expression of *HRG* was observed in advanced lung cancer and is associated with the disease stage (Winiarska et al., 2019). Another study has reported that *HRG* suppresses glioma growth by modulating antitumor immunity through regulating leukocyte differentiation (Roche et al., 2018).

In order to further elucidate the potential function of the CNC8SIG, we performed functional enrichment analysis for genes co-expressed the CNC8SIG. Functional enrichment analysis suggested that genes co-expressed the CNC8SIG are enriched in known cancer-related biological pathways. For example, the dysregulated Wingless-type (Wnt)/β-catenin pathway involved the multistep process of cervical carcinogenesis and could be a candidate as potential biomarker or therapeutic target (Bahrami et al., 2017). The altered expression of genes in the PI3K-Akt signaling pathway has critical roles in tumor initiation, progression and outcomes, including cervical cancer (Zhang et al., 2015). These results indicated that the altered expression of the CNC8SIG participated in broad biological functions associated with metastasis of cervical cancer.

Some limitations of this study existed. First, although predictive performances of the CNC8SIG have been validated in several patients’ cohorts, more independent datasets were needed to validate our findings. Second, the molecular mechanism of the CNC8SIG in the lymph node metastasis and prognosis in cervical cancer should be made in further experimental studies, although *in silico* prediction for the CNC8SIG was performed in this study. Third, our study focused on only mRNAs and lncRNAs. Other non-coding RNA types, such as miRNAs and circRNAs, should be considered in further study.

## Data Availability Statement

Publicly available datasets were analyzed in this study. This data can be found here: Cervical cancer patients used in this study were obtained from the UCSC Xena (http://xena.ucsc.edu), GSE26511 (https://www.ncbi.nlm.nih.gov/geo/query/acc.cgi?acc=GSE26511), and GSE44001 (https://www.ncbi.nlm.nih.gov/geo/query/acc.cgi?acc=GSE44001).

## Author Contributions

YYZ conceived and designed the study. YZ, DS, JS, and NY analyzed the data. YZ and YYZ wrote and revised the manuscript. All authors read and approved the final manuscript.

## Conflict of Interest

The authors declare that the research was conducted in the absence of any commercial or financial relationships that could be construed as a potential conflict of interest.

## Footnotes

1 http://xena.ucsc.edu

2 https://www.ncbi.nlm.nih.gov/geo/query/acc.cgi?acc=GSE26511

3 https://www.ncbi.nlm.nih.gov/geo/query/acc.cgi?acc=GSE44001

4 https://www.gencodegenes.org/#

5 https://david.ncifcrf.gov/home.jsp

## Supplementary Material

Supplementary Table 1 Lists of differentially expressed mRNAs and lncRNAs between cervical cancer patients with and without lymph node metastasis.
